# Deoxypodophyllotoxin Exerts Anti-Cancer Effects on Colorectal Cancer Cells Through Induction of Apoptosis and Suppression of Tumorigenesis

**DOI:** 10.3390/ijms20112612

**Published:** 2019-05-28

**Authors:** Chathurika D. B. Gamage, So-Yeon Park, Yi Yang, Rui Zhou, İsa Taş, Woo Kyun Bae, Kyung Keun Kim, Jung-Hyun Shim, Eunae Kim, Goo Yoon, Hangun Kim

**Affiliations:** 1 College of Pharmacy and Research Institute of Life and Pharmaeutical Sciences, Sunchon National University, 255 Jungang-ro, Sunchon, Jeonnam 57922, Korea; chathurika.gamage@gmail.com (C.D.B.G.); sinbu17@naver.com (S.-Y.P.); yangyi_520@hotmail.com (Y.Y.); zhourui274@hotmail.com (R.Z.); mr.isatas@gmail.com (İ.T.); 2 Department of Internal Medicine, Chonnam National University Medical School, 160 Baekseo-ro, Dong-gu, Gwangju 61469, Korea; drwookyun@chonnam.ac.kr; 3 Department of Pharmacology, Chonnam National University Medical School, 160 Baekseo-ro, Dong-gu, Gwangju 61469, Korea; kimkk@chonnam.ac.kr; 4 College of Pharmacy and Natural Medicine Research Institute, Mokpo National University, 1666 Yeongsan-ro, muan, Jeonnam 58554, Korea; s1004jh@mokpo.ac.kr (J.-H.S.); gyoon@mokpo.ac.kr (G.Y.); 5 College of Pharmacy, Chosun University, 309 Philmun-daero, Dong-gu, Gwangju 61452, Korea; eunaekim@chosun.ac.kr

**Keywords:** deoxypodophyllotoxin, tubulin polymerization, apoptosis, mitotic arrest, tumorigenic potentials, colorectal cancer

## Abstract

Deoxypodophyllotoxin (DPT) is a cyclolignan compound that exerts anti-cancer effects against various types of cancers. DPT induces apoptosis and inhibits the growth of breast, brain, prostate, gastric, lung, and cervical tumors. In this study, we sought to determine the effect of DPT on cell proliferation, apoptosis, motility, and tumorigenesis of three colorectal cancer (CRC) cell lines: HT29, DLD1, and Caco2. DPT inhibited the proliferation of these cells. Specifically, the compound-induced mitotic arrest in CRC cells by destabilizing microtubules and activating the mitochondrial apoptotic pathway via regulation of B-cell lymphoma 2 (Bcl-2) family proteins (increasing Bcl-2 associated X (BAX) and decreasing B-cell lymphoma-extra-large (Bcl-xL)) ultimately led to caspase-mediated apoptosis. In addition, DPT inhibited tumorigenesis in vitro, and in vivo skin xenograft experiments revealed that DPT significantly decreased tumor size and tumor weight. Taken together, our results suggest DPT to be a potent compound that is suitable for further exploration as a novel chemotherapeutic for human CRC.

## 1. Introduction

Colorectal cancer (CRC) has currently been reported as the third-most commonly diagnosed malignancy and as the fourth leading cause of cancer-related deaths in the world [1], and therefore represents a major public health concern. The incidence of CRC exhibits substantial geographical variation: almost 55% of cases occur in developed countries, whereas the mortality rate is higher in the developing world [1].

The treatment strategy for CRC is chosen largely based on the stage of the cancer. The primary forms of treatment are surgery and chemotherapy, which cure approximately 50% of patients. However, recurrence followed by surgery is a major problem in CRC, and often causes the ultimate death [2]. Due to the poor progress of diagnosis and identification of CRC, novel anti-cancer agents are urgently required.

Microtubules are tube-shaped protein polymers consisting of 13 protofilaments, which are in turn composed of heterodimers of α- and β-tubulin [3]. In addition to being a key component of the cytoskeleton, microtubules are associated with a variety of cellular processes, including maintenance of cell shape and motility, intracellular transportation, cell signaling, protein trafficking, and cell division and mitosis. During mitosis, microtubules form highly dynamic mitotic spindles, which are indispensable for the segregation of the chromosomes into the two daughter cells. Disruption of microtubule dynamics during mitosis cause mitotic arrest and eventually apoptosis. Due to the important role that microtubules play in cell motility and mitosis, they are critical targets for the development of anti-cancer drugs [4,5,6].

Microtubule-targeting agents can be classified into two main groups based on their mechanism of action: microtubule-stabilizing or -destabilizing. All microtubule-targeting agents used in cancer therapy act by inducing either mitotic arrest or apoptosis by misdirecting the formation of the mitotic spindle in proliferating tumor cells [7]. Microtubule-stabilizing agents, such as paclitaxel (Taxol) and docetaxel, inhibit microtubule depolymerization by promoting microtubule polymerization. By contrast, microtubule-depolymerizing agents, such as colchicine and vinca alkaloids, inhibit microtubule polymerization and disrupt spindle formation during mitosis [8]. Because disruption of microtubule dynamics tends to inhibit cancer growth, the development of microtubule-targeting drugs has attracted a great deal of interest in the field of cancer chemotherapy.

Caspase-mediated apoptosis is a widely studied mechanism of cell death that plays a critical role in cancer treatment. One process that leads to apoptosis mediated by cascade activation of the caspase is the mitochondrial apoptotic pathway regulated by the B-cell lymphoma 2 (Bcl-2) family of pro-apoptotic and anti-apoptotic proteins [9,10].

To identify more potent anti-cancer agents against CRC cells, we screened the structurally similar anti-cancer agents podophyllotoxin [11], picropodophyllotoxin [12], and deoxypodophyllotoxin (DPT). Among them, podophyllotoxin is the most studied compound, and etoposide and teniposide derived from podophyllotoxin are used clinically as chemotherapeutics in certain types of cancer [13,14]. We found that DPT had the highest cytotoxicity (in the nanomolar range) against CRC cells. DPT is a cyclolignan compound originally derived from extracts of plants used in herbal medicine, including citrus-related plants such as *Podophyllum* (*P.*) *peltatum*, *P. pleianthum*, *P. emodi*, and *Diphylleia grayi* [15]. Previous studies revealed that DPT inhibits growth of prostate, breast, brain, gastric, lung, and cervical cancer cells and induces the cells to undergo apoptosis. In addition, DPT has antiviral, anti-inflammatory, anti–platelet aggregation, and antiallergic properties [16,17,18,19,20,21,22].

In this study with DPT, we focused on the mechanism of action underlying the high cytotoxicity of DPT against CRC cells. First, we found that DPT induces apoptosis in CRC cells by activating the mitochondrial pathway via regulation of Bcl-2 family proteins, Bax and Bcl-xL. Further studies revealed that DPT induces mitotic arrest in CRC cells, leading to apoptosis, as a result of tubulin depolymerization. Moreover, sub-lethal concentrations of DPT inhibited CRC cell migration. Furthermore, DPT suppressed tumorigenesis in vivo in a xenograft mouse model. Our findings confirm that DPT is a potent therapeutic against human CRCs and suggest that this strong apoptosis-inducing agent has the potential to be developed further as an anti-cancer agent.

## 2. Results

### 2.1. DPT Exerted Potent Cytotoxic Effects on Human CRC Cells

**Figure 1:**
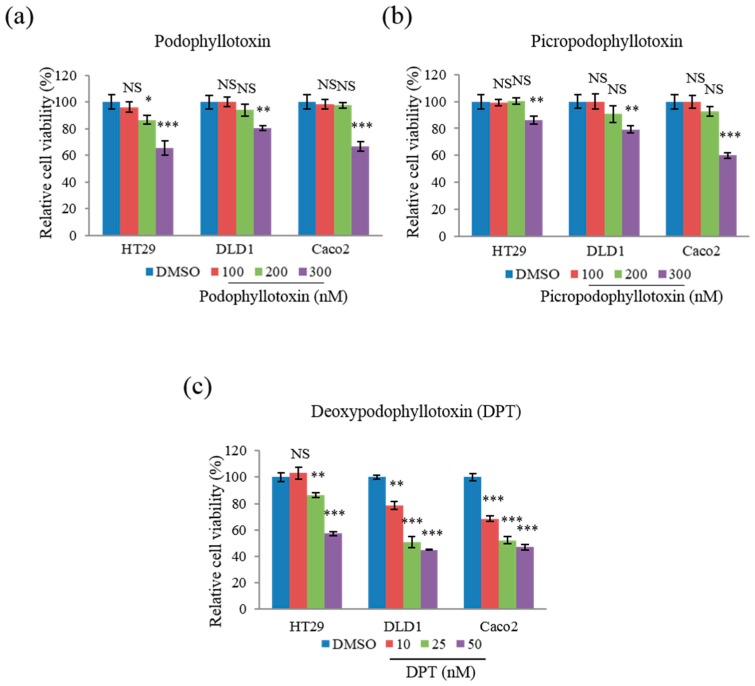
Cytotoxic effects of podophyllotoxin, picropodophyllotoxin, and deoxypodophyllotoxin (DPT) in CRC cells. Cells were treated for 48 h with podophyllotoxin (**a**) and picropodophyllotoxin (**b**) at concentrations from 100 to 300 nM, and DPT (**c**) at concentrations from 10 to 50 nM. Cell viability was measured using an MTT assay. Data represent means ± S.E.M.; *n* = 3. * *p* < 0.05; ** *p* < 0.01; *** *p* < 0.001; NS, no significant difference compared with the DMSO-treated group.

To determine whether DPT has stronger cytotoxic effects than the other compounds, we performed the 3-(4,5-dimethylthiazol-2-yl)-2,5-diphenyltetrazolium bromide (MTT) assay using DPT and two other compounds, podophyllotoxin and picropodophyllotoxin, which have structures similar to those of DPT. This study used three colorectal cancer cell lines, HT29, DLD1, and Caco2, harboring different statuses of the microsatellite instability (MSI) and the mutations of cancer critical genes: HT29 has microsatellite stable (MSS) and mutant *BRAF* and *PIK3CA*; DLD1 has MSI and mutant *KRAS* and *PIK3CA*; and Caco2 has MSS and wild-type *KRAS*, *BRAF*, and *PIK3CA* [23]. At a concentration of 300 nM, podophyllotoxin decreased cell viability by 20–35% (Figure 1a), and picropodophyllotoxin by 15–55% (Figure 1b), in the CRC cell lines HT29, DLD1, and Caco2. DPT had a much stronger cytotoxic effect than the other compounds at low concentrations (10, 25, or 50 µM) (Figure 1c): in all three cell lines, DPT reduced the cell viability by 25–50% at the very low concentration of 50 nM.

The IC50 values (i.e., the dose of DPT that achieved a 50% reduction in viability) for DPT were 23.4, 26.9, and 56.1 nM in DLD1, Caco2, and HT29 cells, respectively. By contrast, podophyllotoxin and picropodophyllotoxin decreased viability by 50% in all three cell lines at concentrations ranging from 300 to 600 nM (Table 1). Together, these results suggest that DPT exerted potent cytotoxic effects against CRC cell lines.

### 2.2. Lethal Concentrations of DPT Induce Apoptosis in CRC Cells

**Figure 2:**
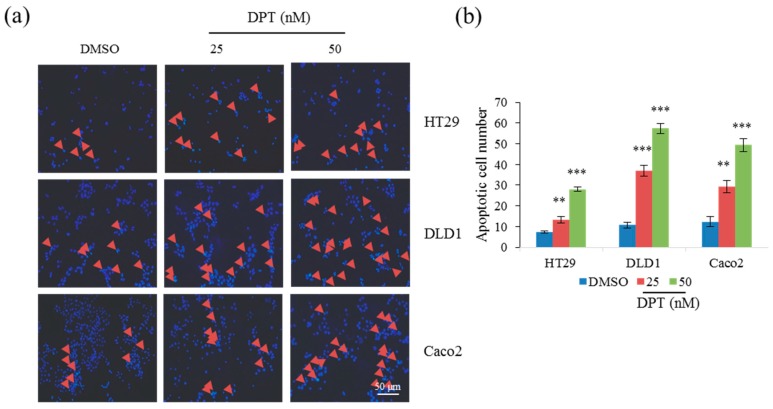

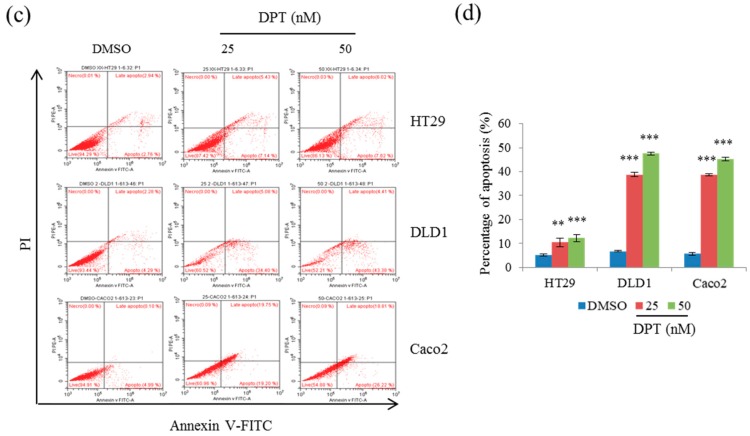
Induction of apoptosis in CRC cells by DPT, as determined by staining with either Hoechst (**a**,**b**) or propidium iodide (PI)/Annexin V–FITC (**c**,**d**). (**a**) Hoechst staining of CRC cells treated with 25 or 50 nM DPT for 12 h. Arrowheads indicate nuclear condensation in cells. (**b**) Quantification of cells with condensed nuclei in Hoechst stained cells treated with 25 or 50 nM DPT. (**c**) PI and Annexin V–FITC staining of CRC cells treated with 25 or 50 nM DPT for 48 h. (**d**) Quantification of apoptosis in PI/Annexin V-FITC stained cells treated with 25 or 50 nM DPT. Results are representative of three experiments. Data represent mean ± S.E.M. ** *p* < 0.01; *** *p* < 0.001 compared with the DMSO-treated group.

To determine whether DPT induces apoptotic cell death, we treated cells with DPT at 25 nM and 50 nM toxic concentrations for 12 h and stained with Hoechst 33258 to observe apoptotic features in nuclear morphology. A large number of CRC cells in initial stage of apoptosis with condense nuclei ware detected among DPT treated cells (Figure 2a). Quantified data showed that the number of apoptotic cells was significantly higher in CRC treated with 50 nM DPT compared to 25 nM DPT (Figure 2b). As DPT treatment for 12 h initiated apoptosis in CRC cells, next, flow cytometric analysis of cells stained with Annexin V–FITC and PI was performed after the treatment with DPT for 48 h. Annexin V binds to cells in early apoptosis, whereas PI stains cells in late apoptosis, as well as dead cells. CRC cells treated with DPT exhibited elevated percentages of both early and late apoptotic cells (Figure 2c). The effect was dose-dependent: quantitative data revealed that cells treated with 50 nM DPT had a higher proportion of apoptotic cells than those treated with 25 nM DPT (Figure 2d). Together, these findings confirm that the cytotoxicity of DPT in CRC cells is due to induction of apoptosis.

### 2.3. DPT Induced Mitotic Arrest Via Destabilization of Microtubules

**Figure 3:**
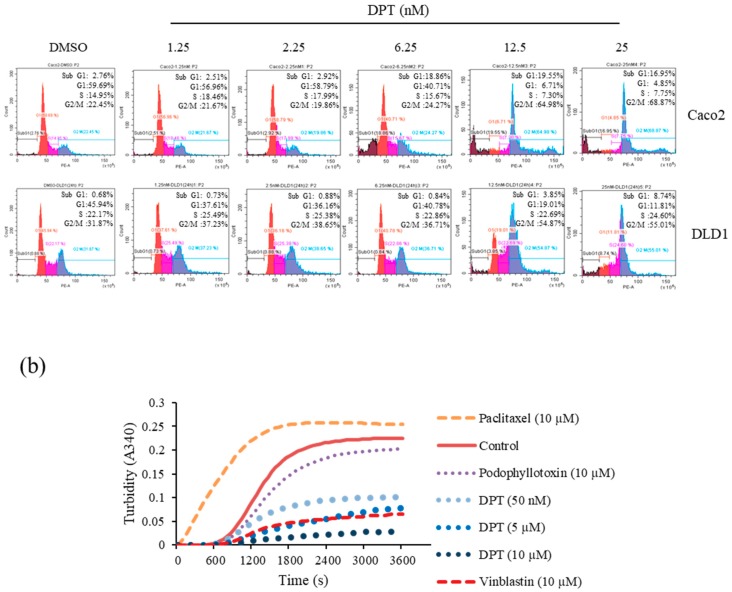
Induction of mitotic arrest in human CRC cells and inhibition of tubulin polymerization by DPT. (**a**) Flow cytometric analysis of the cell-cycle distribution of Caco2 (48 h) and DLD1 (24 h) cells after treatment with DPT at concentrations ranging from nontoxic to toxic (1.25–25 nM). (**b**) Effects of DPT at 50 nM, 5 µM, and 10 µM on the *in vitro* polymerization of purified tubulin. The effect of DPT was examined in a GTP-containing buffer. Results are representative of three experiments.

To investigate the effect of DPT on the cell cycle, we performed flow cytometric cell-cycle profiling. Treatment of Caco2 and DLD1 cells with DPT for 48 h or 24 h, resulted in dose-dependent accumulation of G2/M-phase cells with 4N DNA content and a decrease in G1/S-phase cells (Figure 3a). Cells treated with a lethal concentration of 25 nM DPT exhibited very clear accumulation in the G2/M phase. The dose-dependent increase in the population of cells in the G2/M phase suggested that DPT might induce mitotic arrest.

Because microtubule dynamics play a major role in the mitotic phase of the cell cycle, we performed an in vitro tubulin polymerization assay to confirm the results described above. In mitotic phase, microtubules rearrange to form the mitotic spindle, which supports chromosome segregation and progression through the cell cycle. Disruption of microtubule dynamics by stabilizing or destabilizing microtubules leads to apoptotic death of cancer cells [8,24]. Based on these findings, we compared the effect of DPT on in vitro tubulin polymerization to those of known microtubule-acting agents by measuring the change in turbidity. In the absence of treatment (control), self-assemblage of tubulin heterodimers occurred in a time-dependent manner to form linear tubulin polymers. As expected, the microtubule-stabilizing agent paclitaxel promoted tubulin polymerization, whereas the microtubule-depolymerizing agents vinblastine and podophyllotoxin interfered with tubulin polymerization. Similarly, DPT also interfered with tubulin polymerization in a dose-dependent manner but greater than the others (Figure 3b). Compared with other tubulin depolymerization agents, DPT can destabilize microtubules even at concentrations as low as 50 nM. Thus, these data indicate that destabilization of microtubules by DPT causes mitotic arrest and prevents CRC cells from progressing through the cell cycle.

### 2.4. DPT Induced Cell Death by Regulating B-Cell Lymphoma 2 (Bcl-2) Family Proteins

**Figure 4:**
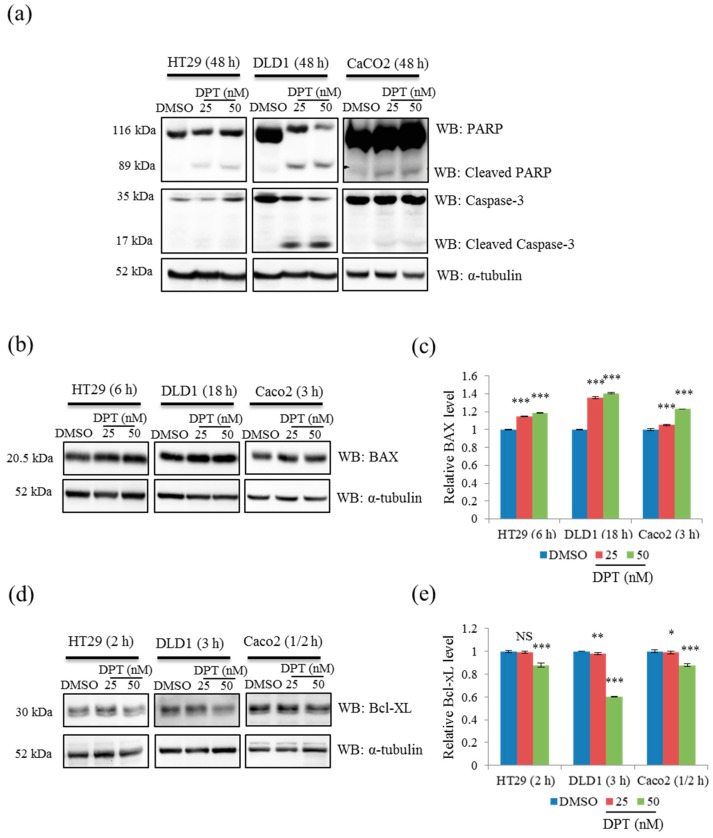
Activation of apoptosis by DPT in CRC cells. (**a**,**b**,**d**) Western blot of poly(ADP-ribose) polymerase (PARP) and caspase-3 (**a**), BAX (**b**), and Bcl-xL (**d**) in cells treated with 25 or 50 nM DPT. (**c**,**e**) Quantification of Bax (**c**) and Bcl-XL (**e**) protein levels in cells treated with DPT. Data represent means ± S.E.M. * *p* < 0.05; ** *p* < 0.01; *** *p* < 0.001; NS, no significant difference compared with the DMSO-treated group.

Next, we performed western blotting to confirm the induction of apoptosis by DPT. Cleaved caspase-3 and cleaved PARP (poly(ADP-ribose) polymerase) were detected in HT29, DLD1, and Caco2 cells after 48 h of treatment with DPT at the lethal concentrations of 25 and 50 nM (Figure 4a). Activation of caspase-3, an effector caspases, led to cleavage of various downstream substrates, including PARP, during apoptosis [10]. In addition, we examined the effect of DPT on the regulation of Bcl-2 family proteins, which function in the mitochondrial intrinsic apoptosis pathway, by detecting changes in the levels of the pro-apoptotic protein Bax and the anti-apoptotic protein Bcl-xL. Western blots revealed that the level of Bax was significantly increased in a dose-dependent fashion via treatment with DPT (Figure 4b,c). By contrast, the level of Bcl-xL was significantly decreased by DPT, also in a dose-dependent manner (Figure 4d,e). These results indicate that DPT can also induce apoptosis by activating the mitochondrial pathway. In light of our previous results showing that DPT induced mitotic arrest in CRC, the subsequent detection of apoptotic proteins (cleaved caspase-3 and PARP) suggested that prolonged mitotic arrest also eventually leads to apoptosis. Taken together, these results demonstrate that lethal concentrations of DPT cause apoptosis in CRC cells by inducing mitotic arrest, as well as activating the mitochondrial apoptosis pathway.

### 2.5. Sub-Lethal Concentrations of DPT Inhibited Migration, but not Invasion, of CRC Cells

**Figure 5:**
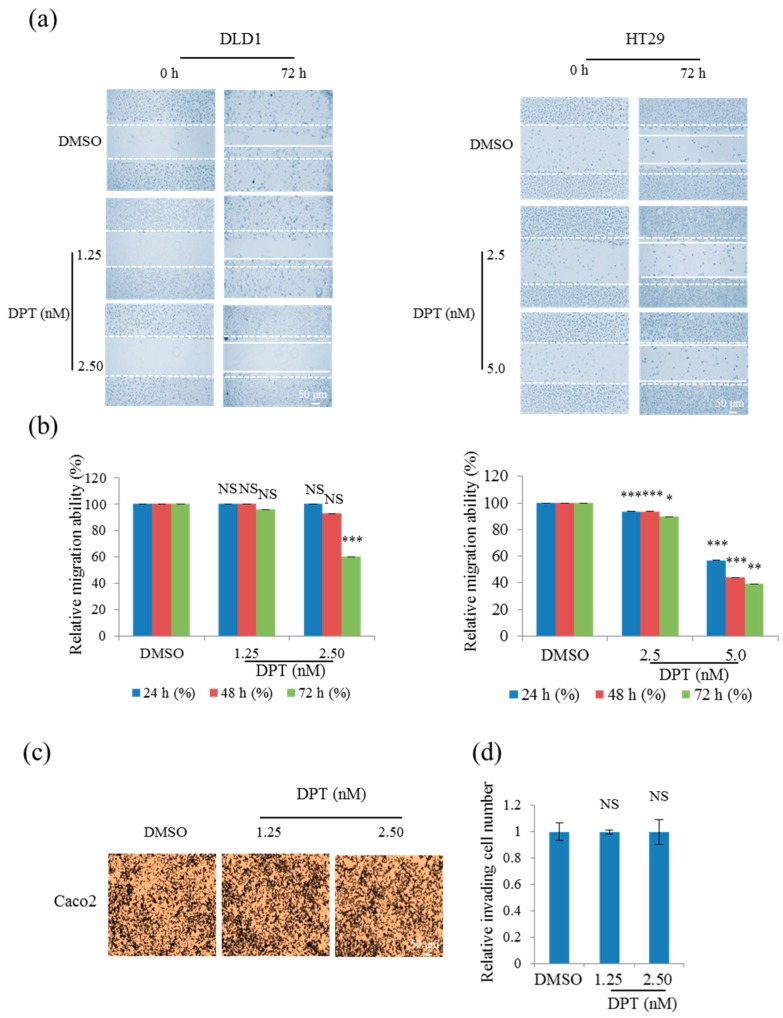
Inhibition of CRC cell motility by DPT. (**a**,**b**) Migration assay of DLD1 and HT29 cells treated with 1.25, 2.5, or 5 nM DPT (**a**), and quantitative analysis of wound length (b). (**c**,**d**) Invasion assays in Caco2 cells treated with 1.25 or 2.5 nM DPT; (**d**) shows quantitation of invading cell numbers in each group. Representative images from three independent experiments are shown in (**c**). Data represent means ± S.E.M. * *p* < 0.05; ** *p* < 0.01; *** *p* < 0.001; NS, no significant difference relative to DMSO-treated Caco2 cells.

Cancer cell migration and invasion cause tumor metastasis. Hence, we performed wound-healing and invasion assays to determine whether DPT affects migration and invasion, respectively. To this end, we exposed CRC cells to 5% or 10% of the lethal concentrations of DPT (sub-lethal concentration; i.e., 1.25 or 2.5 nM). The results revealed that DPT treatment significantly inhibited the CRC cell migration (Figure 5a,b), but had no effect on the invasion by Caco2 cells (Figure 5c,d). Overall, these results suggest that sub-lethal concentrations of DPT inhibited the migratory potential of CRC cells, probably by suppressing microtubule dynamics.

### 2.6. Sub-Lethal Concentrations of DPT Inhibited Tumorigenicity of CRC Cells

**Figure 6:**
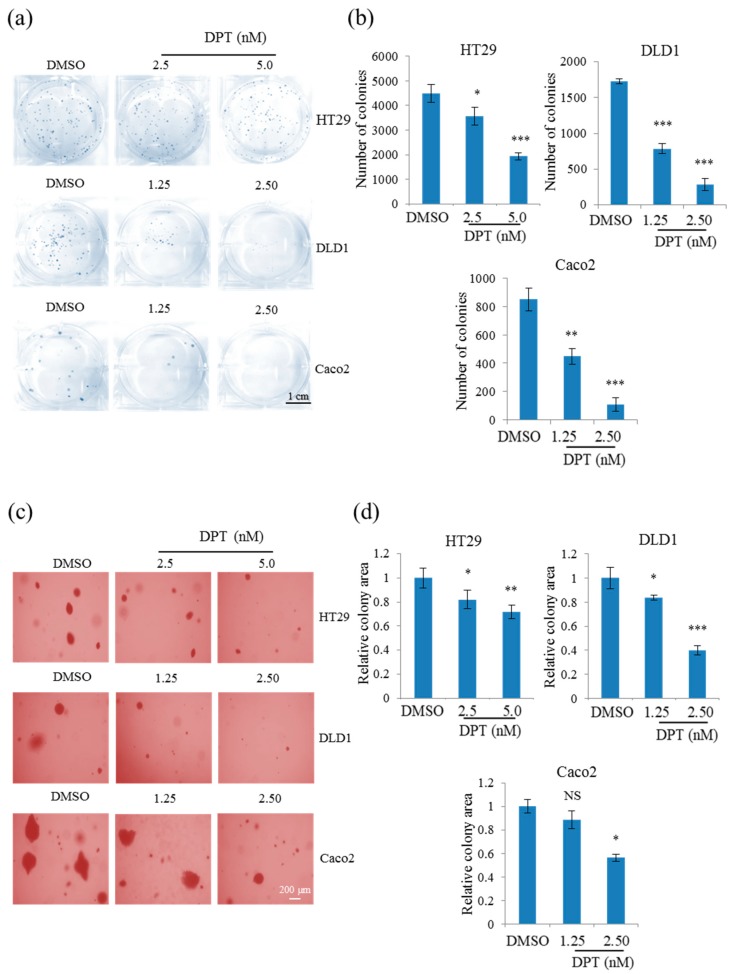
Inhibition of proliferation and anchorage-independent growth of HT29, DLD1, and Caco2 cells using sub-lethal concentrations of DPT. (**a**,**b**) Clonogenic assay of HT29, DLD1, and Caco2 cells treated with DPT (**a**) and quantification of colony number in each group (b). (**c**,**d**) Soft-agar colony-formation assays of HT29, DLD1, and Caco2 cells treated with DPT (**c**), and quantification of percent colony area in each group (**d**). Representative images are shown from three independent experiments. Data represent means ± S.E.M.; * *p* < 0.05; ** *p* < 0.01; *** *p* < 0.001; NS, no significant difference compared with the DMSO-treated group.

To further explore the anti-cancer activity of DPT, we tested the in vitro tumorigenicity of HT29, DLD1, and Caco2 cells exposed to concentrations of DPT that did not induce cytotoxicity. Clonogenic assays of CRC cells treated with sub-lethal concentrations of DPT (DLD1 and Caco2: 1.25 or 2.5 nM; HT29: 2.5 or 5 nM) revealed a significant reduction in the number of colonies, showing that the proliferation of the cell was inhibited at these concentrations (Figure 6a,b). A soft-agar colony-formation assay was performed to determine whether sub-lethal concentrations of DPT inhibit anchorage-independent growth of CRC cells (Figure 6c,d). Colony formation of CRC cells on soft agar was significantly decreased by treatment with DPT in a dose-dependent manner. These results demonstrate that DPT has anti-tumorigenic activity at sub-lethal concentrations.

### 2.7. DPT Inhibited Tumor Growth in a Xenograft Mouse Model

**Figure 7:**
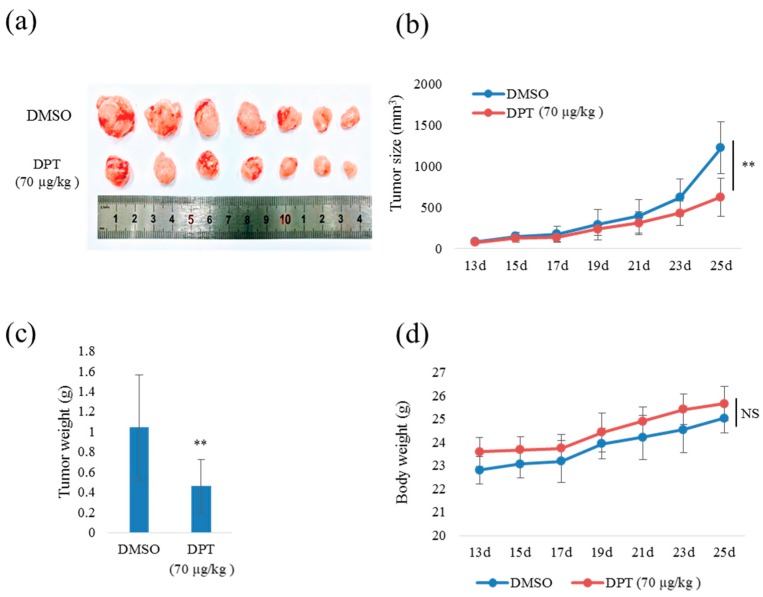
Inhibition of in vivo tumorigenesis by DPT in a xenograft mouse model. (**a**) Images of tumor tissues, representative of seven mice from the DPT-treated group and DMSO-treated control group. (**b**,**c**) Quantitative analysis of isolated tumor size (b) and tumor weight (c) for each group. (**d**) Body weight of the control and DPT-treated mice. Results are reported as means ± S.E.M.; ** *p* < 0.01; NS, no significant difference compared with the DMSO-treated group.

To evaluate the anti-tumorigenic effect of DPT in vivo, we monitored tumor growth in a skin tumor xenograft mouse model. CT26, a murine colorectal carcinoma cell line from a BALB/c mouse, was used to stablish a xenograft mouse model as it is a syngeneic cell line with a similar IC50 value (23.6 nM) against DPT treatment with human CRC cell lines HT29, DLD1, and Caco2. Thirteen days after the injection of CT26 cells, tumor-bearing mice were intraperitoneally administered with DMSO (control) or DPT (70 µg/kg) once every 2 days for ≈2 weeks. The dosage amount was set based on the IC50 value of DPT on CT26 cells and the referenced literatures [25,26]. The results revealed that tumor size decreased over time in DPT-treated mice relative to the control group (*n* = 7) (Figure 7a,b). After the mice were sacrificed, tumor weight was significantly smaller in the DPT-treated group than in the control group (Figure 7c), although body weights did not differ between groups (Figure 7d), indicating that the dose of DPT administered did not exert widespread cellular cytotoxicity. Taken together, these results demonstrate that DPT suppressed tumor growth in a mouse xenograft model in vivo.

## 3. Discussion

DPT is a phytochemical compound that exerts anti-cancer effects against multiple kinds of cancers: it induces G2/M cell-cycle arrest in HeLa cervical cancer cells and caspase-mediated apoptosis in DU-145 prostate cancer cells, glioblastoma U-87 MG and SF126 cells, and SGC-7901 gastric cancer cells, [15,17,18,21]. Furthermore, DPT induces apoptosis of MDA-MB-231 breast cancer cells through the extrinsic pathway and triggers necroptosis in NCI-H460 non–small cell lung cancer cells [16,20]. DPT also has some in vivo anti-tumor effects against SGC-7901 gastric cancer cells and MDA-MB-231 cells, as well as H460 non–small cell lung cancer cells [18,19,20]. In this study, we investigated the anti-cancer activity of DPT against three CRC cell lines: HT29, DLD1, and Caco2. The results revealed that DPT had stronger cytotoxic effects in these cell lines than podophyllotoxin and picropodophyllotoxin. This is the first report of the anti-cancer activity of DPT against CRC. Our findings show that DPT exerts cytotoxicity against CRC cells by inducing apoptosis at lethal concentrations, inhibits migration and tumorigenesis by these cells at sub-lethal concentrations, and inhibits tumorigenesis in vitro and in vivo.

Apoptosis plays a significant role in fighting cancer. Accordingly, it is a popular target for cancer treatment strategies, and is considered to be one of the most promising ways to inhibit cancers [27]. In this study, we focused on the suppression of CRC via induction of cell apoptosis. Hoechst staining of CRC cells showed the initiation of the apoptosis process via nuclear condensation after the treatment with DPT for 12 h and the number of cells with condensed nuclei was increased dose-dependently. The Annexin V–PI double-staining assay revealed that the rate of apoptosis in HT29, DLD1, and Caco2 cells increased significantly after 48 h of DPT treatment. Cell-cycle analyses confirmed that DPT strongly induced the accumulation of mitotic cells in both Caco2 and DLD1 cells, suggesting that DPT induces cell-cycle arrest in M phase. Further investigations revealed that DPT destabilizes microtubules by inhibiting tubulin polymerization. Induction of apoptosis using a 48 h DPT treatment was confirmed through western blot analysis of caspase and PARP cleavage. In addition, DPT treatment upregulated the pro-apoptotic protein Bax and downregulated the anti-apoptotic protein Bcl-xL.

Targeting microtubules is one of the most important strategies for anti-cancer therapy because of their critical role in mitosis [7,8]. During M phase, microtubules of the interphase cytoskeleton depolymerize, and tubulin repolymerizes to form the mitotic spindle. Then, the duplicated chromosomes are localized and attached to the spindle for segregation. This process requires highly coordinated microtubule dynamics [6,24]. Known microtubule destabilizers, such as vinca alkaloids, colchicine, and podophyllotoxin, cause the arrest of the cell-cycle at mitosis by disrupting microtubule dynamics that prevent the formation of the mitotic spindle and eventually lead to apoptosis [28]. Our flow cytometric cell-cycle analysis revealed that DPT induced G2/M-phase arrest. In a tubulin polymerization assay, DPT exerted effects similar to those of known microtubule destabilizers vinblastine and podophyllotoxin, even at a low concentration (50 nM). Western blot results confirmed the activation of apoptosis, as reflected by the cleavage of caspase-3 and PARP. Accordingly, the effect of DPT was similar to those of known microtubule destabilizers: arrest of cell-cycle at mitosis, eventually leading to apoptosis.

Caspase family proteins are key regulators of the initiation and execution of apoptosis. Among them, caspase-3 is an effector caspase, which is responsible for the actual cleavages of intracellular proteins, such as lamin and PARP, resulting in apoptotic cell death of many cell types. Caspase-3 is activated as a result of the cytoplasmic release of pro-apoptotic protein cytochrome c [18,27]. Bcl-2 family proteins—which consist of pro-apoptotic BH3-only proteins, pro-apoptotic proteins (Bax, Bak), and anti-apoptotic proteins (Bcl-xL)—also play pivotal roles in the early steps of apoptotic cell death [29]. Activation of Bax leads to the release of cytosolic cytochrome c, which is responsible for caspase activation. The pro-apoptotic protein Bax is activated in two main ways: direct binding of a BH3-only protein to Bax or binding of a BH3-only protein to anti-apoptotic proteins such as Bcl-xL. In the latter indirect mode, Bax and Bak become activated after being displaced from anti-apoptotic proteins by BH3-only proteins [30]. Activated Bax oligomerizes to form pores in the mitochondrial outer membrane, resulting in the release of cytochrome c. Released cytosolic cytochrome c leads to caspase activation and subsequent cell death. Expression of caspase-3 and Bcl-2 family proteins is intimately associated with the intrinsic mitochondrial pathway [9,10,27]. The observed cleavage of caspase-3 and PARP, upregulation of Bax, and downregulation of Bcl-xL following DPT treatment suggested that DPT can also induce apoptosis in CRC cells through the intrinsic mitochondrial pathway. Moreover, evasion of apoptosis is mainly regulated by three mechanisms: 1) disruption of the balance between pro-apoptotic and anti-apoptotic proteins, 2) reduction in caspase function, and 3) impairment of death receptor signaling [27]. Our findings indicate that DPT regulates two of these mechanisms in HT29, DLD1, and Caco2 cells by maintaining the balance between pro-apoptotic and anti-apoptotic proteins by increasing and decreasing the levels of pro-apoptotic and anti-apoptotic proteins, respectively, and increasing caspase activity. In addition, our findings indicate that the effect of DPT on the expression of apoptosis-related proteins is time-, dose-, and cell line–dependent. These observations corroborate previous reports showing that DPT causes apoptosis by inducing G2/M cell-cycle arrest in cervical cancer [18,21] and activating the caspase-3–mediated intrinsic mitochondrial pathway in prostate and brain cancers [15,17]. DPT induced apoptosis in human CRC cells at lethal concentrations ranging from 25 to 50 nM. The ability of DPT to cause apoptosis via two pathways may explain its high cytotoxicity in CRC cells at nanomolar concentrations.

Bcl-2 family proteins involved in apoptosis play significant roles in tumor development or tumor regression. Pro-apoptotic proteins inhibit tumorigenesis by inducing apoptosis. Conversely, anti-apoptotic proteins promote tumorigenesis by inhibiting apoptosis [31]. Our soft-agar colony-formation and clonogenic assay results revealed that DPT inhibited CRC tumorigenesis in vitro in a dose-dependent manner at sub-lethal concentrations. These results suggest that upregulation of BAX and downregulation of Bcl-xL inhibited CRC tumorigenesis in vitro. Our findings in the xenograft mouse model confirmed the suppression of CRC tumorigenesis in vivo. In those experiments, DPT decreased the growth of CT26 cells in mice, but did not affect body weight, indicating that DPT suppressed the growth of CRC xenografts without eliciting general toxicity at the dose we used (70 µg/kg). As pharmacokinetic parameters for DPT are not reported yet and we did not measure the actual DPT concentration in plasma or distribution to tumor tissue, the dosage amount of DPT needs to be further optimized. Our finding is consistent with those of previous studies showing that DPT can suppress the tumorigenesis in vivo in gastric, breast, and non–small cell lung cancer [18,19,20]. Sufficient mechanistic understanding and the potential clinical usefulness of DPT, including its potential toxicity toward cancer stem cells, should be further elucidated.

## 4. Materials and Methods

### 4.1. Cell Culture and Reagents

The CRC cell lines HT29, DLD1, and Caco2 were purchased from the Korean Cell Line Bank (Seoul, South Korea). Authentications of the cells used in the study were performed by a commercial service (Korean Cell Line Bank) via short tandem repeat profiling. Cells were cultured in either RPMI 1640 or DMEM culture medium (GenDEPOT, Katy, TX, USA) supplemented with 10% fetal bovine serum (GenDEPOT) and 1% penicillin–streptomycin solution. Cells were cultured in a humidified atmosphere at 37 °C in 5% CO_2_. Podophyllotoxin and picropodophyllotoxin were purchased from Sigma-Aldrich (St. Louis, MO, USA). DPT was prepared as previously described and provided by Prof. Goo Yoon [32].

### 4.2. MTT Assay

Cells ((1.5–5) × 10^3^ cells/well) were seeded on 96-well plates, grown overnight, and then treated with 10–50 nM DPT for 48 h. After a 4 h incubation with MTT at 37 °C, the cells were lysed with 150 µL of DMSO (Sigma-Aldrich), and absorbance was measured at 570 nm using a spectrophotometer (Bio Tek Instruments, Winooskim, VT, USA).

### 4.3. Hoechst Staining

CRC cells were seeded in a 12-well plate at a density of 2 × 10^5^ cells/well, allowed to attach overnight, and treated with 25 nM and 50 nM DPT for 12 h. Cells were washed three times with phosphate-buffered saline (PBS), fixed in 4% paraformaldehyde for 15 min, washed again with PBS, and permeabilized in 0.1% Triton X-100 (Sigma-Aldrich) for 30 min, followed up incubation with Hoechst 33258 (Sigma-Aldrich) for 1 h in dark at room temperature. Stained cells were mounted onto glass coverslips and analyzed using a Nikon Eclipse 400 fluorescence microscope (Nikon Instech Co., Ltd.,Kawasaki, Japan).

### 4.4. Flow Cytometric Analysis

Cells were seeded into six-well plates at a density of 2 × 10^5^ cells/well, cultured overnight, treated with 25 or 50 nM DPT for 48 h, trypsinized, and washed with ice-cold PBS. All cells were resuspended in 100 µL of 1× binding buffer containing 5–10 µL of 50 µg/mL propidium iodide (PI; BD Biosciences, San Jose, CA, USA) and 1–5 µL of Annexin V–FITC (BD Biosciences, San Jose, CA, USA), incubated for 15 min in the dark, and analyzed using flow cytometry on a CytoFLEX instrument (Beckman Coulter Life Sciences, Indianapolis, IN, USA).

### 4.5. Flow Cytometric Analysis for Cell Cycle

Caco2 and DLD1 cells were seeded in six-well plates at a density of 2 × 10^5^ cells/well, cultured overnight, treated with 1.25–25 nM DPT for 24 or 48 h, trypsinized, and washed with FACS wash solution. First trypsin solution was added and incubated for 10 min and then RNase inhibitor was added and incubated for 10 min at room temperature. Next, the samples were centrifuged, the supernatants were removed, and the pellets were resuspended in 100 mL of 4 mg/mL PI (Sigma-Aldrich, St. Louis, MO, USA) and incubated for 2 h in the dark at 4 °C. Flow cytometry was performed with a CytoFLEX instrument (Beckman Coulter Life Sciences, Indianapolis, IN, USA).

### 4.6. Tubulin Polymerization Assay

Tubulin polymerization was analyzed using the Tubulin Polymerization Assay Kit (Cytoskeleton, Inc., Denver, CO, USA). In brief, tubulin proteins (>99% pure) were suspended at a final concentration of 3.0 mg/mL in ice-cold TP buffer (80 mM PIPES (pH 6.9), 2 mM MgCl_2_, 0.5 mM EGTA, 1.0 mM GTP, 8.5% glycerol). Then, the tubulin solution was incubated at 37 °C with a general tubulin buffer alone (control) or with DPT (50 nM, final concentration of 5 or 10 µM). Paclitaxel and vinblastine (final concentration, 10 µM) were used as positive controls for the promotion and inhibition of polymerization, respectively. Tubulin polymerization was measured by continuously monitoring the change in turbidity at 340 nm (Bio Tek Instruments).

### 4.7. Western Blotting

Cells were treated with 25 or 50 nM DPT for the indicated times and lysed; the extracted protein was separated using SDS-PAGE. The density of bands was measured using the Multi-Gauge 3.0 (Fujifilm, Tokyo, Japan) software, and their relative density was obtained based on the density of the α-tubulin bands in each sample. Then, values were expressed as arbitrary densitometric units corresponding to the signal intensity. Antibodies against PARP, caspase-3, Bax, Bcl-xL, and α-tubulin were purchased from Cell Signaling Technology (Danvers, MA, USA).

### 4.8. Clonogenic Assay

HT29, DLD1, and Caco2 cells were washed, trypsinized, and resuspended in RPMI 1640. Then, cells were seeded into six-well plates at 500 cells/well in 2.5 mL of RPMI 1640/DMEM and incubated to allow attachment. After 48 h treatment, media containing DPT was replaced with fresh medium and then incubated for 12 days. Colonies were fixed by 4% paraformaldehyde, stained with 0.5% crystal violet, and quantified under a microscope. The plating efficiency of untreated cells and the survival fraction of treated cells were then determined (*n* = 3).

### 4.9. Soft-Agar Colony-Formation Assay

HT29, DLD1, and Caco2 cells were suspended in soft agar (3 × 10^3^ cells/1 mL of 0.35% agarose in RPMI/DMEM complete medium), plated on 1 mL of solidified agar (0.5% agarose in RPMI/DMEM complete medium) into 12-well plates, and cultured for 3 weeks. Cells were fed twice per week with cell culture media containing DPT (1.25, 2.5, or 5 nM) or DMSO (0.01%). The surface areas of the colonies were measured in randomly selected microscope fields in each plate using the IMT i-Solution FL-Auto software (IMT i-Solution Inc., Northampton, NJ, USA). To measure the relative area of the colony, pixel amounts of colony areas were normalized. Data represent the means of three experiments.

### 4.10. Wound Healing Assay

Cells were plated at a density of (2.5–3) × 10^5^ cells/well and grown overnight to confluence; the resultant monolayers were scratched to create a wound. Then, the cells were washed twice and incubated in RPMI 1640/DMEM culture medium supplemented with 2% FBS containing 0, 1.25, 2.5, or 5 nM DPT. To obtain relative migration ability, photographs of cells at 0, 24, 48, and 72 h after wounding were taken to measure the width of the wound. The distance migrated by the cells was measured as the difference in distance between the edges of the wound between the time points. An average of five wound distances for each sample was taken to determine the average rate of migration at a given DPT concentration. The migration rate was determined according to the following formula: migration rate [%] = {(width t_1_ [mm] − width t_2_ [mm])/width t_1_} × 100% (t_1_–Initiating time, t_2_–Ending time).

### 4.11. Invasion Assay

The invasion of tumor cells was analyzed using Transwell chambers (Corning Costar, Corning, NY, USA). Upper chambers contained polycarbonate membranes with 8 µm pores coated with 1% gelatin. Caco2 cells were seeded in culture medium containing 0.2% bovine serum albumin (BSA) at 2.5 × 10^5^ cells/well in the upper compartment of the chamber. The lower compartment of the chamber was filled with a culture medium containing 0.2% BSA and 1 mg/mL fibronectin as a chemo-attractant. Cells were cultured for 24 h in the absence or presence of 1.25 or 2.5 nM DPT. The upper chambers were fixed and stained using the Diff Quick kit (Sysmex, Kobe, Japan). Invading cells were quantified under a light microscope in five randomly selected fields. Experiments were performed in triplicate. Results are expressed as the mean of migrating cells per high-power field.

### 4.12. In Vivo Mouse Xenograft Model

Five-week-old, pathogen-free conditioned male BALB/c nude mice were obtained from OrientBio (Gyeonggi-do, Korea). The Sunchon National University Research Institutional Animal Care & Use Committee approved the animal experiments.

To establish skin tumor xenografts, CT26 cells were trypsinized and suspended in PBS. The cell suspension (1 × 10^5^ cells in 0.1 mL of PBS/mouse) was injected subcutaneously into the right flank of each mouse (*n* = 7 for each group). After the tumor volume reached 100 mm^3^, mice were intraperitoneally administered with vehicle (DMSO/PBS) or DPT at a dose of 70 µg/kg of body weight once every 2 days. Body weight and caliper measurements were taken every 2 days. Tumor volumes were calculated using the following formula: volume in mm^3^ = 0.5 × L × W^2^, where L is length and W is width. Tumors were monitored regularly and allowed to grow until the tumor volume in the control mice reached ≈1500 mm^3^. Thereafter, mice were sacrificed, and the tumors were collected and weighed.

### 4.13. Statistical Analysis

All experiments were performed multiple times. Data are expressed as means ± standard error of the mean. All statistical analyses were performed using IBM Statistical Package for SocialScience (SPSS) version 22. Treatment effects were determined using one-way ANOVA post-hoc analysis. Unless indicated otherwise, a *p*-value < 0.05 was considered significant.

## 5. Conclusions

DPT exerted potent anti-cancer activity against human CRC cells in vitro and in vivo. It caused apoptosis by inducing G2/M cell-cycle arrest, as well as activating the intrinsic mitochondrial pathway, and could inhibit CRC tumorigenesis both in vitro and in vivo. At lethal concentrations, DPT caused cytotoxicity by inducing apoptosis. At sub-lethal concentrations, DPT inhibited cell migration and suppressed tumorigenesis. Similar to etoposide and teniposide derived from podophyllotoxin, which are clinically used for chemotherapeutics in certain types of cancer, our results provide an evidentiary basis for the use of DPT as a potential anti-cancer reagent that could be used for further research and treatment of human CRC.

## Figures and Tables

**Table 1:** IC50 value of CRC cells treated with podophyllotoxin, picropodophyllotoxin, and deoxypodophyllotoxin (DPT).

Compounds	IC50 (nM)		
	HT29	DLD1	Caco2
Podophyllotoxin	694.7	461.8	380.5
Picropodophyllotoxin	532.1	580.1	334.0
Deoxypodophyllotoxin (DPT)	56.1	23.4	26.9

HT29, DLD1, and Caco2 cells were seeded in 96-well plates at (2–5) × 10^3^ cells/well and incubated with the indicated concentrations of podophyllotoxin, picropodophyllotoxin, and DPT for 48 h. The MTT assay was performed to determine the cytotoxicity of compounds, and IC50 was assessed using the SPSS statistical software. All values represent means ± SDs from triplicate experiments.

## Abbreviations

CRC	Colorectal cancer
DPT	Deoxypodophyllotoxin
DMSO	Dimethylsulfoxide
PARP	Poly(ADP-ribose) polymerase
Bcl-xL	B-cell lymphoma-extra large
PBS	Phosphate-buffered saline
